# Plexiform Schwannoma of the nasal tip: surgical approach

**DOI:** 10.1590/S1808-86942012000300022

**Published:** 2015-10-14

**Authors:** Renata Caroline Mendonça Ferraz, Juliana Antoniolli Duarte, Reginaldo Raimundo Fujita, Shirlei Shizue Nagata Pignatari

**Affiliations:** a MD (first-year ENT resident at EPM-UNIFESP).; b MD (second-year ENT resident at EPM-UNIFESP).; c MD (PhD and Associate Professor at EPM-UNIFESP).; d MD (PhD and Associate Professor at EPM-UNIFESP). Escola Paulista de Medicina - Universidade Federal de São Paulo (EPM-UNIFESP).

**Keywords:** schwann cells, nose, neurofibroma

## INTRODUCTION

Schwannomas and neurofibromas are the most frequently seen neurogenic tumors, and both present a plexiform variation. Men and women between the ages of 30 and 60 are equally affected. Schwannomas account for a significant share of head and neck tumors (25-45%), 4% of which are found in the nasal cavity and paranasal sinuses^1^, ^2^, ^3^.

Schwannomas are usually solitary encapsulated tumors located peripherally. They are somewhat soft to the touch and are made up exclusively of Schwann cells^4^, ^5^. Schwannomas originate from the sympathetic, parasympathetic, or sensorial nerves^1^. Plexiform tumors infiltrate more and have poorly defined margins^1^, ^2^.

This case report aims to discuss the ways to surgically approach the rare manifestations of nasal tip plexiform Schwannomas.

## CASE PRESENTATION

**Figure 1:**
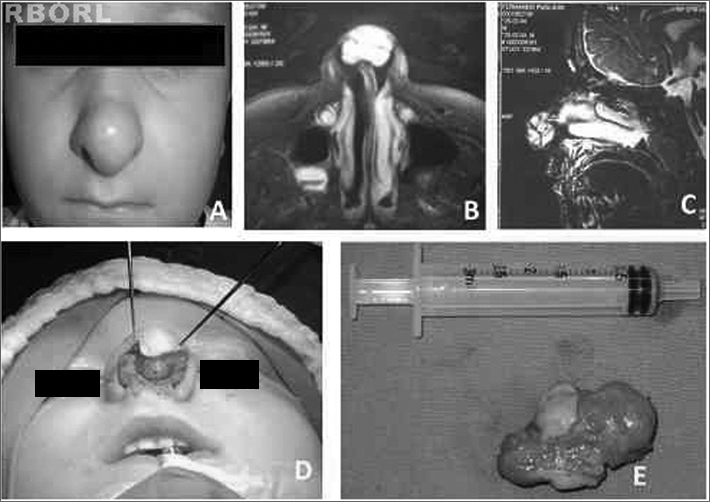
A) Nasal tip tumor. B) Preoperative MRI; axial view showing hypersignal on T2. C) MRI sagittal view showing hypersignal on T2. D) Exo-rhinoplasty with Rethi's incision. E) Tumor specimen with approximately 2.5 cm.

F.P., 6, male, came to our service to have a deformity in his nasal tip looked at. The deformity had been present for two years (Figure 1-A), after the patient fell and bruised his nose. Since then he started having intermittent episodes of epistaxis unaccompanied by nasal obstruction.

Physical examination revealed a well-defined fibroelastic tumor in the patient's nasal tip. Anterior rhinoscopy showed cleared nasal cavities. MRI scans showed a well-defined 2.5-cm tumor involving the alar cartilages with hypersignal on T2 (Figures 1-B and C).

We opted to perform exo-rhinoplasty using Rethi's incision (Figure1-D). This technique comprises a series or marginal incisions on the caudal borders of the alar cartilages connected to a columellar incision. Skin, subcutaneous cell tissue, and perichondrium are moved to expose both the nasal tip and nasal dorsum, thus facilitating the removal of the tumor. Margins were not removed due to the location of the tumor.

Pathology and immunohistochemistry tests of surgical specimens were a match for plexiform Schwannoma and positive for proteins vimentin and S-100.

The patient came back to our service in the second year of his follow-up with another nasal tip tumor and complaining of nosebleed episodes that had been bothering him for the past three months. MRI scans showed images similar to the ones produced prior. The patient underwent surgery to treat his recurring tumor. Pathology tests confirmed he had a plexiform Schwannoma.

## DISCUSSION

Four cases of nasal tip Schwannoma have been reported in the literature, three benign and none of the plexiform subtype.

In terms of pathology test findings, Schwannomas may be divided into Antoni types A and B depending for higher and lower cell counts respectively. Immunohistochemistry is important for etiological diagnosis, and a test positive for protein S-100 is required^1^.

The most common complaint is nasal obstruction followed by epistaxis. Our patient had only nosebleeds. Previous history of trauma can be used in the differential diagnosis against post-trauma nasal neurofibroma^1^, ^3^, ^6^.

CT and MRI scans are useful in planning for surgery^1^.

Surgery is the only treatment, as these tumors resist to radiotherapy and chemotherapy is not an option^1^.

The surgical approach may vary. Larger tumors involving the paranasal sinuses and skull base are preferably treated via the Caldwell-Luc procedure, degloving or lateral rhinotomy. Smaller and intranasal tumors may be approached endoscopically. Rhinoplasty is the option of treatment for nasal tip tumors. Disfiguring incisions are unnecessary, given the low risk for malignant degeneration^2^, ^4^. The location of the tumor was used as criterion to pick exo-rhinoplasty as the chosen procedure for our patient.

Recurring tumors are rare (2%) when removal is complete, and are particularly associated with neurofibromatosis. Plexiform tumors present even lower risk levels despite their more infiltrative pattern of occurrence, which by its turn may hamper the removal of the tumor in one procedure and require additional procedures^2^. Despite the recurring tumor, our patient had no signs of neurofibromatosis. There are no cases of plexiform Schwannomas reported in the literature. As this is the first report, we cannot describe precisely the tumor recurrence patterns.

## CONCLUSION

Nasal tip plexiform Schwannomas are rare. The microscopic nature of their branches makes it harder for one to find the nerve of origin. Plexiform Schwannomas are not encapsulated. They may be located diffusely and are possibly more associated with local recurrence. Broad disfiguring excisions are unnecessary given the low rates of malignant degeneration. Exo-rhinoplasty provided for a good cosmetic result on this nasal tip tumor.
